# *Rosmarinus officinalis* L. as Fascinating Source of Potential Anticancer Agents Targeting Aromatase and COX-2: An Overview

**DOI:** 10.3390/molecules30081733

**Published:** 2025-04-12

**Authors:** Adriana Gargano, Ilario Greco, Carmine Lupia, Stefano Alcaro, Francesca Alessandra Ambrosio

**Affiliations:** 1 Dipartimento di Scienze della Salute, Università degli Studi “Magna Græcia” di Catanzaro, Campus “S. Venuta”, Viale Europa, 88100 Catanzaro, Italy; a.gargano@unicz.it (A.G.); ilario.greco@studenti.unicz.it (I.G.); ambrosio@unicz.it (F.A.A.); 2 Associazione CRISEA—Centro di Ricerca e Servizi Avanzati per l’Innovazione Rurale, Loc. Condoleo, 88055 Belcastro, Italy; 3 Mediterranean Ethnobotanical Conservatory, 88054 Sersale, Italy; studiolupiacarmine@libero.it; 4 National Etnobotanical Conservatory, Castelluccio Superiore, 85040 Potenza, Italy; 5 Net4Science Academic Spin-Off, Università “Magna Græcia” of Catanzaro, Campus “S. Venuta”, Viale Europa, 88100 Catanzaro, Italy

**Keywords:** cancer, natural compounds, Mediterranean area, *Rosmarinus officinalis* L., aromatase, cyclooxygenase-2

## Abstract

Cancer is the second leading cause of death in the world, with scientific evidence indicating that the enzymes aromatase and cyclooxygenase 2 are upregulated in several types of cancer. Over the past 30 years, natural compounds have played a crucial role in cancer chemotherapy, and to date, many phytocompounds have been reported to interact with these enzymes, inhibiting their activity. Notably, several phytocompounds found in *Rosmarinus officinalis* L., a medicinal plant native to the Mediterranean region and cultivated around the world, have shown the ability to interact with these enzymes. This review examines the role of the main compounds contained in *Rosmarinus officinalis L*. as potential anticancer agents acting on aromatase and cyclooxygenase-2.

## 1. Introduction

*Rosmarinus officinalis* L., rosemary, is an aromatic plant belonging to the Lamiaceae family and native to the Mediterranean area; however, it can be found all over the world [1,2]. Rosemary’s historical significance as a plant is particularly fascinating, as it has been recognized as one of the most well-known plants since ancient times.

For instance, ancient Egyptians used rosemary-based oils and lotions to protect themselves from desert heat and extreme temperatures, and the Egyptian pharaohs’ journeys to the underworld were perfumed with rosemary bunches placed in their tombs [3]. The evidence of the arrival of rosemary in China dates back to around 220 BC [3].

In reference to its ability to tone up a tired body, via a topical application, Dioscorides (40–90 BC) named rosemary *Libanotis coronaria* (pre-Linnaean nomenclature) [3], and De Materia Medica, the precursor of contemporary pharmacopeias, states that rosemary was used as an insecticide and in aromatherapy to perfume rooms. Additionally, ointments containing rosemary flowers and leaves macerated in olive oil were used as a vulnerary to treat wounds and as a remedy for joint discomfort by the founders of ancient medicine such as Hippocrates, Avicenna, and Galen [3].

The genus *Rosmarinus* includes several species. Notably, *Rosmarinus officinalis* L. (rosemary) is a species of Mediterranean origin and cultivated worldwide for its aromatic properties. It is a small perennial shrub with fragrant evergreen needle-shaped leaves and white, pink, purple, or blue flowers [4].

The fresh and dried leaves are often used in traditional Mediterranean cooking and folk medicine. Chemical constituents include bitter principle, resin, tannic acid, volatile oils, and flavonoids [3,5]. The volatile oil consists of borneol, bornyl acetate, camphene, cineol, pinene, and camphor.

Nowadays, it is well known for its hepatoprotective, antimicrobial, antithrombotic, anti-atherosclerotic, diuretic, antidiabetic, hypolipidemic, hypocholesterolemic, anti-inflammatory, antioxidant, and anticancer activity [6,7,8,9]. The essential oil is useful for arthritis, gout, muscle pain, neuralgia, wounds, and premature baldness. Species of the genus *Rosmarinus* are also used in veterinary medicine for their anthelmintic and acaricidal uses, for example, against Varroa destructor, which commonly affects the Western honeybee (*Apis mellifera*) [10].

Regarding anticancer activity, it has been reported that the bioactive compounds of rosemary have either direct or indirect epigenetic targets in cancer chemoprevention and chemotherapy. Accordingly, rosemary extracts and their isolated components show inhibitory effects on the growth of breast, liver, prostate, lung, and leukemia cells [11,12,13].

**Figure 1:**
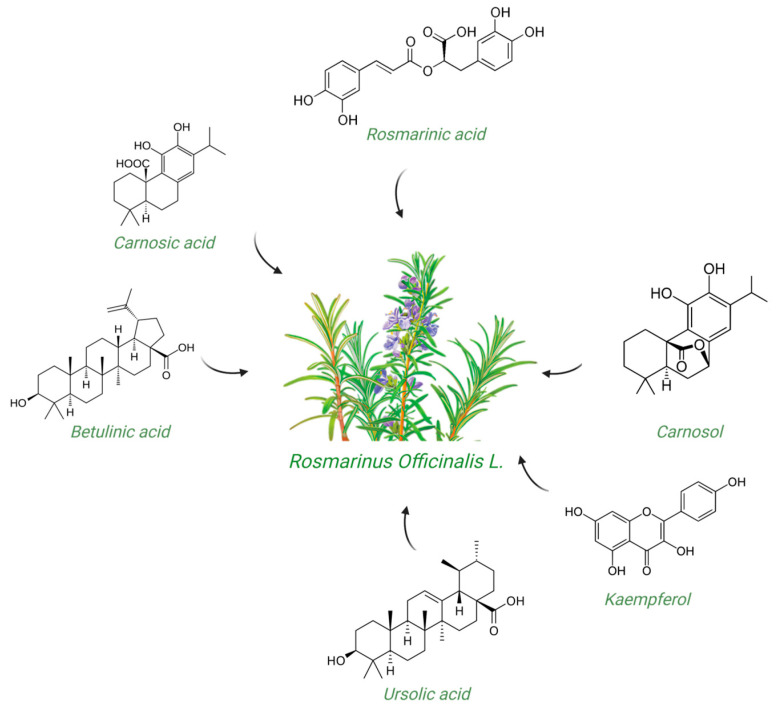
Two-dimensional representation of compounds contained in *Rosmarinus officinalis* L. discussed in this review.

The biological activities of *Rosmarinus officinalis* L. have been associated with the presence of pharmacologically active constituents belonging to phenolic diterpenes and triterpenes; phenolic acids; and flavonoids such as carnosic acid, carnosol, rosmarinic acid, betulinic acid, ursolic acid, and kaempferol (Figure 1) [2,7,14]. Thus, rosemary represents an exceptionally rich source of bioactive compounds [11,13,14,15].

Cancer is a multifactorial disease, and despite numerous discoveries over time, most of its types remain incurable [16,17]. By 2020, in fact, 19 million new cases and 10 million deaths were reported worldwide [18], and it is estimated that at least one out of every two people will be diagnosed with a type of cancer by the age of 85 and that there has been a two-fold increase in mortality in women and a three-fold increase in men over the past 40 years [19].

To date, more than 200 types of cancer are known; the most prevalent are breast, colorectal, lung, prostate, liver, stomach, endometrial, thyroid, and the different types of leukemia [20], and each of these presents different characteristics among the various forms of each class, starting with etiology, risk factors, and the genetic component.

The altered activity of various enzymes plays a critical role in the advancement of cancer, influencing processes such as cell growth, angiogenesis, apoptosis, and metastasis progression. In this context, pivotal roles are attributed to enzymes such as aromatase and cyclooxygenase-2 (COX-2) that contribute significantly to the development and progression of cancer [21,22,23,24,25,26,27].

Recognizing their significant contributions provides avenues for targeted therapeutic interventions to modulate these enzyme activities and potentially impede cancer development and progression [28,29,30,31,32,33].

Thus, the purpose of this review is to summarize the anticancer activity of some of the bioactive constituents of *Rosmarinus officinalis* L., with particular attention on carnosic acid (CA), rosmarinic acid (RA), carnosol (CS), betulinic acid (BA), oleanolic acid (OA), ursolic acid (UA), rosmanol (RO), and kaempferol (KA), highlighting their abilities to interfere with at least one of the mentioned targets.

### 1.1. Aromatase

Aromatase, also known as estrogen synthase, is a member of the cytochrome P450 monooxygenase superfamily. It is the only enzyme in vertebrates known to catalyze, with high specificity, the synthesis of estrogens from their androgenic precursors through the demethylation of androgen carbon 19-producing 18-carbon phenolic estrogens [34,35].

**Figure 2:**
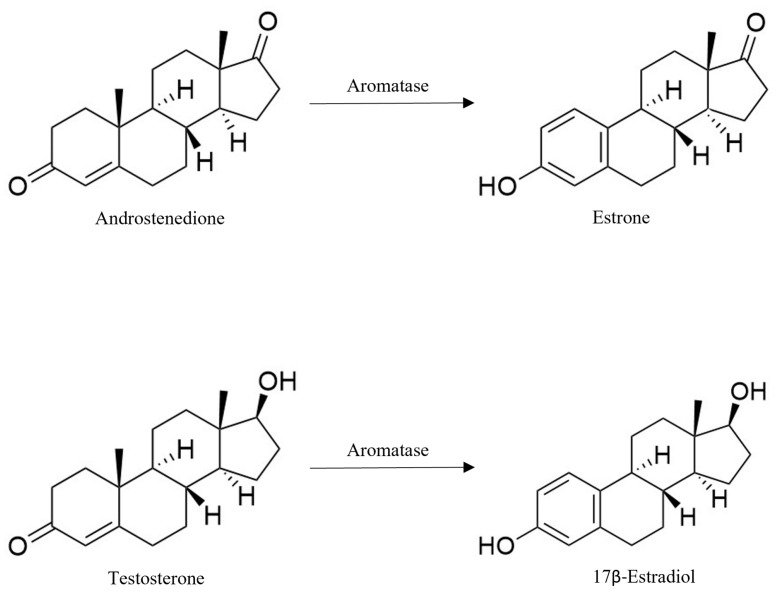
Biosynthesis of estrogen by aromatase. Created with ChemDraw 23.1.2 software [39].

In detail, aromatase with high specificity converts androstenedione and testosterone into estrone (E1) and 17β-estradiol (E2), respectively, through the aromatization of the A-ring (Figure 2). Moreover, other androgens with the same backbone, such as the ones with 15α/16α hydroxylation, are also aromatized by this enzyme [36,37,38].

**Figure 3:**
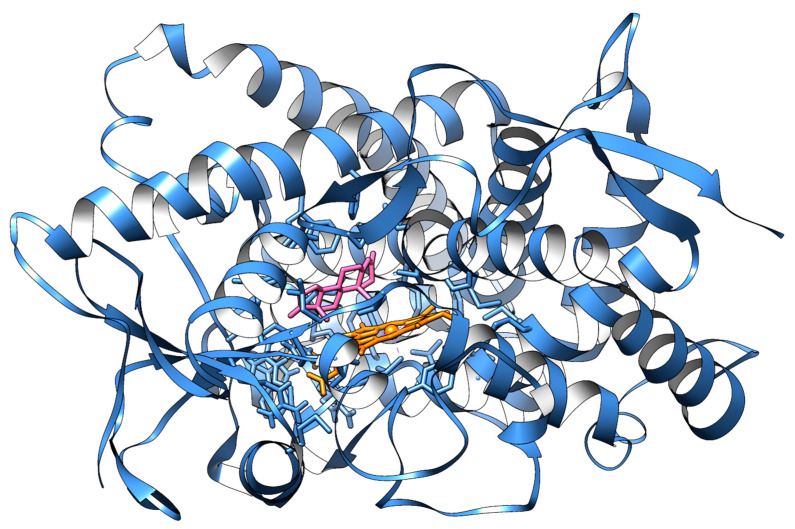
Three-dimensional representation of aromatase in complex with 4-androstene-3,17-dione. The enzyme is depicted as a slate cartoon; the heme group, 4-androstene-3,17-dione, and key residues of the binding site are represented as orange, pink, and slate sticks, respectively. Atomic coordinates were obtained from PDB model 3S79 [34]; the figure was built using Chimera 1.18 software [41].

Aromatase is a 57.9 kDa integral membrane protein of the endoplasmic reticulum, anchored to the membrane by its N-terminal transmembrane domain. The functional human enzyme consists of 503 amino acids organized into twelve α-helices, ten β-strands, and an Fe-heme group (Figure 3) [34,36,38,40]. Specifically, aromatase is localized in the plasma membrane of the endoplasmic reticulum of estrogen-producing cells, such as adipose tissue, skin, and skeletal muscle, and plays a role in development, reproduction, sexual differentiation, behavior, bone and lipid metabolism, and brain function [35,37,40].

Estrogens play a key role in the regulation of important physiological functions, such as regulating the menstrual cycle, modulating bone density, and maintaining vessels and skin. Furthermore, higher levels of estrogen, especially 17-β-estradiol (E2), are involved in the physiopathology of hormone-dependent cancers such as breast, ovary, and endometrial cancer [24]. Considering the crucial role of aromatase in catalyzing the biosynthesis of estrogens from androgens, its inhibition has become the standard treatment for postmenopausal estrogen-dependent breast cancers [34,35,36,37,40].

To date, three generations of aromatase inhibitors (AIs) have been approved by the Food and Drug Administration (FDA). The first generation of AIs, which included aminoglutethimide and testolactone, acted as a model for the later generations of AIs, which are characterized by better selectivity and lower toxicity. Fadrozole and formestane are classified as second-generation AIs. Fadrozole contains an imidazole group, and it is more selective and potent than aminoglutethimide, but unfortunately, it affects the biosynthesis of corticosterone, progesterone, and aldosterone. Formestane is a steroidal analog, and it was the first selective AI to reach clinical trials in the 1990s [36]. Lastly, the third generation of AIs comprises the triazole derivatives anastrozole and letrozole and one steroidal analog, exemestane, and they are recommended by the FDA as first-line drugs for the treatment of breast carcinoma [36].

Although AIs represent the gold standard for endocrine therapy, treatment with AIs may sometimes be not sufficient or compromised due to the development of therapeutic resistance and/or a lack of potency; thus, there is a constant need for the development of new potential drugs able to interfere with aromatase activity characterized by reduced toxicity and side effects.

### 1.2. Cyclooxygenase-2

**Figure 4:**
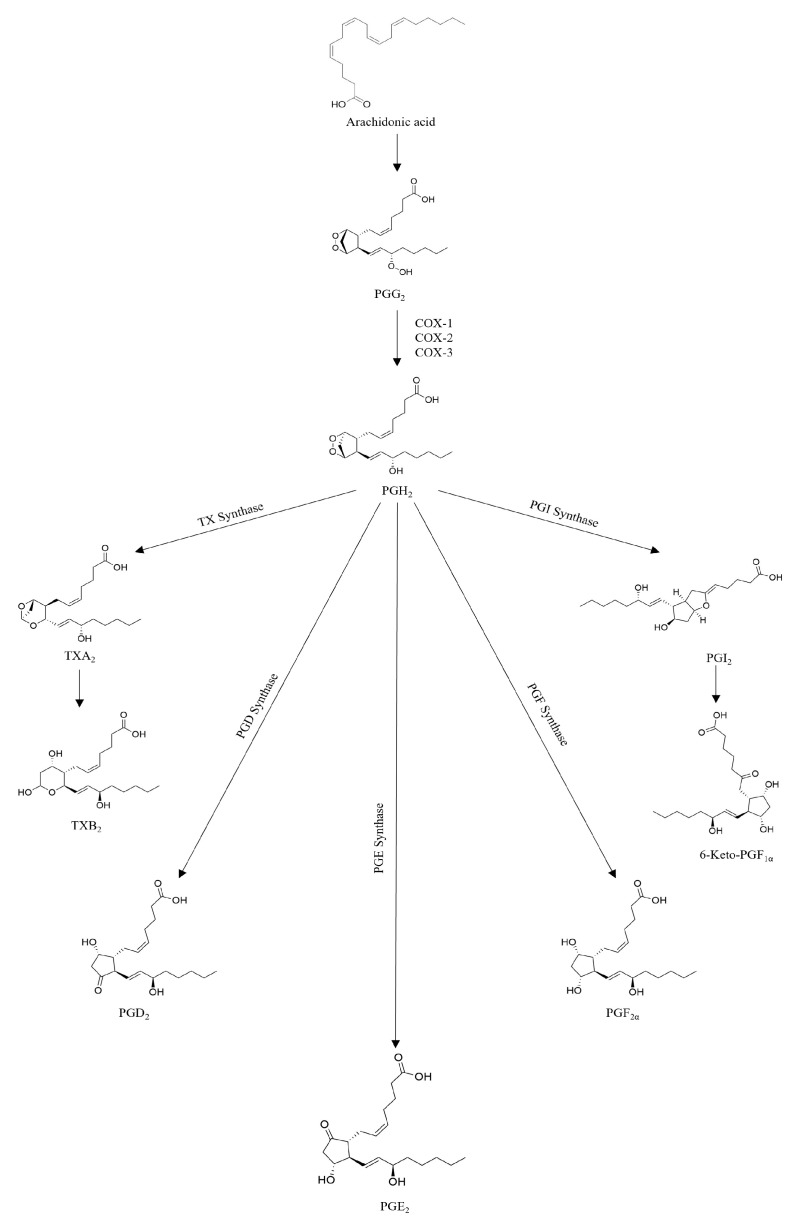
The arachidonic acid cascade. Created with ChemDraw 23.1.2 software [39].

The cyclooxygenases are iron-containing enzymes located at the luminal side of the endoplasmic reticulum and the nuclear membrane that catalyze prostaglandin biosynthesis from arachidonic acid [42,43] (Figure 4). Three COX isoforms, cyclooxygenase-1 (COX1), cyclooxygenase-2 (COX-2), and cyclooxygenase-3 (COX-3), are known. COX-1 is a constitutive ubiquitously isoenzyme, and the prostaglandins produced by COX-1 have protective effects on the gastrointestinal tract [44]. COX-3 can be considered a variant of COX-1, mainly within the central nervous system [42], and COX-2 is an inducible enzyme that acts as the most important source of prostaglandins, so it is always regarded as a pathologic enzyme [43,44].

COX-2, or prostaglandin endoperoxide synthase-2 (PGES-2), is a membrane-bound heme-containing glycoprotein, inducible isoform, that catalyzes the initial step of arachidonic acid’s metabolic transformation into prostanoids (Figure 4) [45,46].

COX-2 is detectable in certain tissues due to a stimulus connected to the anti-inflammatory response; in fact, it can be rapidly upregulated in response to growth factors and cytokines. It is responsible for pain, inflammation, tumorigenesis, proliferation, apoptosis, metastasis, reproductive processes, and circulatory homeostasis.

**Figure 5:**
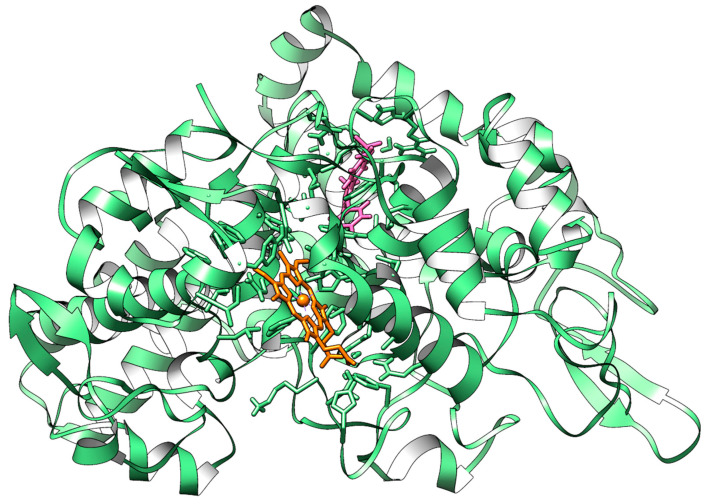
Three-dimensional representation of COX-2 in complex with Rofecoxib. The enzyme is depicted as a green cartoon; the heme group, Rofecoxib, and key residues are represented as orange, pink, and green carbon sticks, respectively. Atomic coordinates were obtained from PDB model 5KIR [47]; the figure was built using the Chimera 1.18 software [41].

From a structural point of view, COX-2 is a homodimeric protein of 604 amino acids and presents several functional domains within the N-terminus and C-terminus of each monomer that regulate COX-2’s cellular localization, membrane anchoring, and catalytic activity [45]. The COX-2 globular catalytic domain constitutes the bulk of the protein and is formed by two distinct interconnected lobes that contain the cyclooxygenase active site and the peroxidase active site, which are separated by the heme prosthetic group (Figure 5) [45].

It is widely known that COX-2 is overexpressed in different types of cancers, such as pancreatic, breast, stomach, and lung carcinoma, as well as in several hematological malignancies. Thus, the implications of COX-2 in tumorigenesis have opened a new frontier in cancer treatment [45,48].

Indeed, nonsteroidal anti-inflammatory drugs (NSAIDs) are frequently used for the management of cancer-related pain and inflammation [48].

Interestingly, the selective COX-2 inhibitor, celecoxib, has shown anticancer properties against some cancers, such as ovarian cancer and adenomas [48]. Furthermore, COX-2 inhibitors have also shown the ability to overcome multidrug resistance, reducing the expression of efflux pumps [48]. Therefore, according to the pivotal and characteristic role of COX-2 in tumorigenesis, it has emerged as a novel possible target for the development of new anticancer drugs [45,48].

## 2. *Rosmarinus officinalis* L.: A Focus on Some of the Most Active Phytocompounds

### 2.1. Carnosic Acid

Carnosic acid (CA) (Table 1) is a natural chemical compound that was initially discovered in *Salvia officinalis* L. and subsequently found in various plants, including *Rosmarinus officinalis* L., *Oreganum*, *Thymus*, *Lepechinia*, and *Ocimum*. *Rosmarinus officinalis* L. is particularly recognized as a rich source of carnosic acid, constituting 4–10% of the weight basis of air-dried leaves [49]. The variability in content is influenced by factors such as variety, climate, environmental conditions, and nutrition [50,51,52]. Carnosic acid is predominantly present in the leaves, petals, and sepals of the *Rosmarinus officinalis* L. plant [52]. Its abundance in these plant parts makes *Rosmarinus officinalis* L. a key source for extracting this compound, which is known for its various biological activities and potential health benefits (Table 1).

By modulating the activity of the COX-2 enzyme, carnosic acid can potentially lead to anticancer effects. In their study, Barni et al. [60] demonstrated that CA inhibits the proliferation and migration capacity of human colorectal cancer cells in three different cell lines: Caco-2 (p53^m^), LoVo (p53^wt^), and HT29 (p53^wt^). The authors proved that CA downregulates the expression of COX-2 at both the mRNA and protein levels. The reduction in COX-2 protein expression was significant, approximately 2–3-fold, and displayed a dose-dependent inhibition of Caco-2 cell migration. Notably, a strong inhibitory effect on COX-2 expression at both the protein and mRNA levels was observed at 96 µM of CA, suggesting that CA could act as a COX-2 inhibitor. Importantly, COX-1 mRNA levels remained unaffected by CA [60].

In 2022, Khella et al. [56] evaluated the anticancer activity of CA in breast cancer cells (MCF-7) and colon cancer cells (Caco-2) by measuring the cell viability, apoptosis rate, and gene expression of key biomarkers such as GCLC, COX-2, and BCL-2. First, in their work, the authors, in order to overcome the challenges of the low water solubility and poor bioavailability of CA, proposed a novel formulation involving the encapsulation of CA in nanoparticles. Then, the antitumor activity of the encapsulated CA was evaluated by measuring the cell viability, apoptosis rate, and gene expression of GCLC, COX-2, and BCL-2 in MCF-7 and Caco-2 [56]. This work revealed a downregulation of COX-2 and BCL-2 expression and an upregulation of GCLC expression. The impact of CA on the expression of GCLC, COX-2, and BCL-2 may help explain the mechanism behind its antitumor activity [56].

### 2.2. Carnosol

Carnosol (Table 2), first isolated from *Salvia pachyphylla Epling ex Munz*, is also found in *Rosmarinus officinalis* L. Carnosol is an ortho-diphenolic diterpene that contains an abietane carbon skeleton with hydroxyl groups at positions C-11 and C-12 and a lactone moiety across the B ring (Table 2) [64]. Notably, carnosol is a naturally occurring polyphenol that is produced by the oxidative degradation of carnosic acid [54,64]. Carnosol possesses a spectrum of biological effects that have been harnessed in both ancient and modern medicine (Table 2).

In their study, Subbaramaiah et al. reported the ability of carnosol to inhibit COX-2 expression in human mammary epithelial cells and the phorbol ester (PMA)-mediated induction of activator protein-1 (AP-1) activity [68]. In detail, in their study, the authors attributed the inhibition of COX-2 induction to the blocking of PKC signaling and, thus, to the binding of AP-1 to the CRE of the COX-2 promoter [68].

### 2.3. Rosmarinic Acid

**Figure 6:**
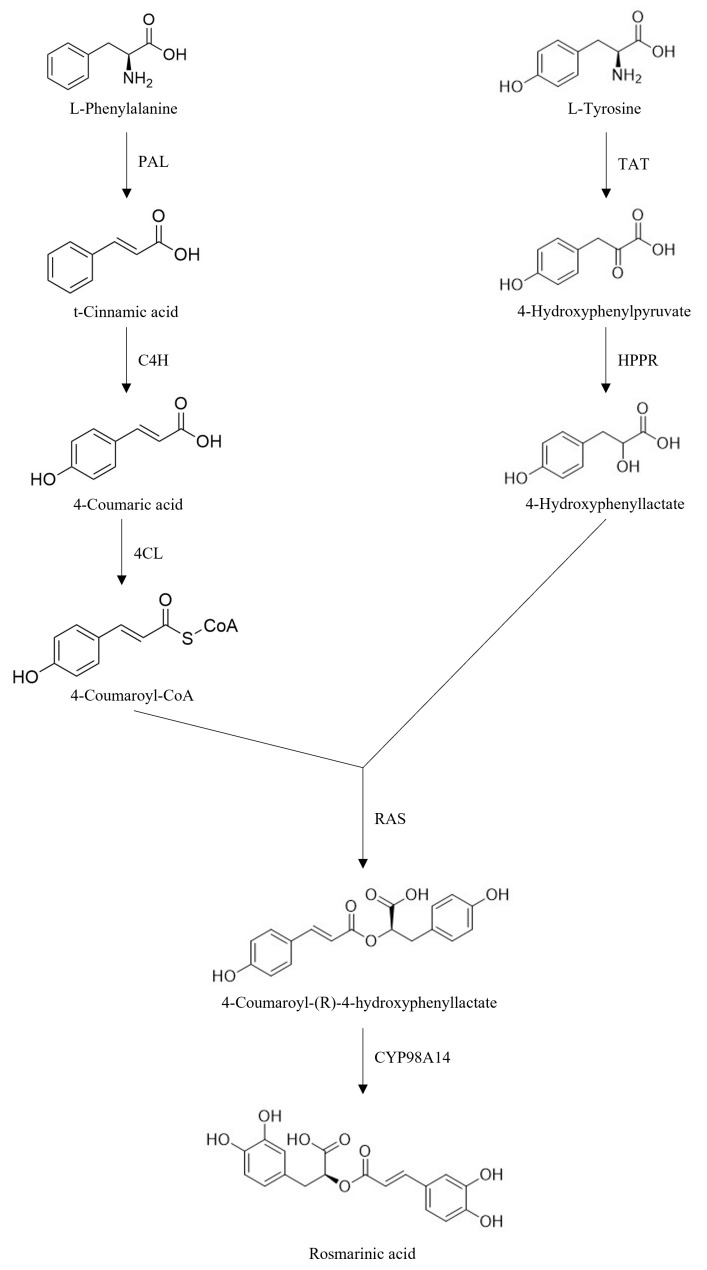
Biosynthetic pathway of rosmarinic acid. (PAL, phenylalanine ammonia-lyase; C4H, cinnamic acid 4 hydrolase; 4CL, 4-coumaryl-CoA ligase; TAT, tyrosine amminotransferase; HPPR, hydroxyphenylpiruvate reductase; RAS, rosmarinic acid synthase; CYP98A14, cytocrome P450-dependent monooxygenase).

Rosmarinic acid (RA) (Table 3) is a water-soluble phenolic compound found abundantly in medicinal plants such as *Rosmarinus officinalis* L., *Origanum vulgare*, *Salvia officinalis*, *Melissa officinalis*, *Origanum majorana*, and *Mentha spicata* belonging to the Lamiaceae family [71]. RA was first isolated as a pure compound by two Italian chemists, Scarpati and Oriente (1958), who named the compound rosmarinic acid according to the plant it was isolated from, *Rosmarinus officinalis*. Additionally, it is present in traditional Chinese medicine plants like *Perilla frutescens* (L.) *Britton* and *Salvia miltiorrhiza Bunge* [72]. It has been shown that, in plants, two amino acids are separately involved in the RA biosynthesis pathways (Figure 6) [71,72].

Rosmarinic acid presents a wide variety of biological activities, as reported in Table 3. In their study, Scheckel et al. [80] examined the effects of RA on COX-2 expression in colon and breast cancer cells, as well as in non-malignant mammary epithelial cells. They reported that RA may exert anti-inflammatory and anticarcinogenic effects by antagonizing the AP-1-dependent activation of COX-2 gene expression. In detail, the authors reported that the cotreatment of HT-29 colon cancer cells with RA reduced TPA-induced COX-2 promoter activity and protein levels; furthermore, RA antagonized the AP-1-dependent activation of COX-2 transcription [80]. Thus, given the role of COX-2 in inflammation and carcinogenesis and the role of AP-1 in proliferation and transformation, the ability of RA to regulate AP-1 activity and COX-2 gene expression implies that RA might serve as a promising preventative agent against COX-2 activation, both in cancerous and nonmalignant mammary epithelial cells.

In 2015, Karthikkumar et al. [79] investigated the potential anticarcinogenic effects of RA in 1,2-dimethylhydrazine (DMH)-induced rat colon carcinogenesis, in which the expression of COX-2 mRNA was upregulated in the colon mucosa. The findings revealed that RA supplementation played a crucial role in downregulating COX-2 expression; in fact, after supplementing DMH-exposed rats with RA, the expression of COX-2 was downregulated in the entire treatment period regimen as compared to unsupplemented DMH-treated rats [79].

In a study conducted by Tao et al. [81], the authors demonstrated the ability of RA and other phenolcarboxylic acids to target COX-2 and, therefore, exert potential antitumor activity. Specifically, the authors performed computational modeling studies highlighting the ability of RA to interact with the enzyme with a good theoretical binding affinity, establishing several interactions with the key residues of the COX-2 binding site. The experimental data further validated the theoretical prediction, revealing that RA effectively inhibits COX-2 activity in the micromolar range and reduces A549 cell proliferation in non-small cell lung cancer (NSCLC) [81].

### 2.4. Betulinic Acid

Betulinic acid (BA) (Table 4) is a hydrophobic pentacyclic lupane-type triterpene found in *Rosmarinus officinalis* L. and various plant families, including *Platanaceae*, *Dilleniaceae*, *Rhamnaceae*, and *Rosaceae* [93,94].

Like other phytochemicals isolated from *Rosmarinus officinalis* L., betulinic acid manifests a range of biological activities, including anti-inflammatory, antimalarial, antiviral, and anticancer properties (Table 4).

BA has excellent biological activities, and its anticancer properties especially deserve attention. In 2021, Ferreira et al. reported the protective effect of BA on doxorubicin (DXR)-induced genotoxicity and 1,2-dimethylhydrazine (DMH)-induced colon carcinogenesis in rodents [95]. The authors investigated the anticarcinogenic activity of BA, highlighting its chemoprotective effect related to a reduction in the level of COX-2 and PCNA expression. In detail, their study underlines that BA modulates the activation of the NF-kB complex, inhibiting COX-2 and consequently blocking cell proliferation [95]. These findings suggest that BA could be a promising compound in anticancer therapies, particularly for colorectal cancer.

### 2.5. Ursolic Acid

Ursolic acid (UA) (Table 5), a phytocomponent of *Rosmarinus officinalis* L., was initially discovered in the epicuticular waxes of apples and later found in various medicinal plants, including *Origanum majorana*, *Ilex paraguariensis*, *Origanum vulgare*, *Salvia officinalis*, *Thymus vulgaris*, *Lavandula angustifolia*, *Eucalyptus*, *Sambucus nigra*, *Crataegus* spp., and *Coffea arabica*, as well as the wax layers of numerous edible fruits [102].

UA can exist in either a free acid form or as an aglycone of saponins [103]. Structurally, UA—3β-hydroxy-urs-12-en-28oic acid—(Table 5) is a pentacyclic triterpenoid with a molecular weight of 456.7 g/mol [102].

*Rosmarinus officinalis* L. is rich in this compound, and it possesses anticancer, anti-inflammatory, antioxidant, antimicrobial, antiviral, antidiabetic, and neuroprotective properties (Table 5). Notably, UA has aroused great interest because of its anticancer properties, exerted through the modulation of proliferation, apoptosis, metastasis, and angiogenesis via various mechanisms [13,104].

**Table 5:** Two-dimensional structure and biological activity of ursolic acid.

Ursolic Acid		
2D	Biological Activity	References
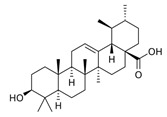	Anticancer	[105,106,107,108,109,110]
	Anti-inflammatory	[111,112,113,114]
	Antidiabetic	[115,116]
	Hypolipidemic	[116,117]
	Hepatoprotective	[118]

Liu et al. [119], in their study, reported the ability of UA to downregulate the expression of COX-2, leading to a reduction in the proliferation of a hepatocellular carcinoma cell line. Through their work, the authors proved that UA possesses anticancer properties, particularly through the inhibition of COX-2, leading the HepG2 cancer cells into an over-oxidative environment, causing apoptosis, and retarding cell proliferation [119].

The structural resemblance between UA and androstenedione suggests that UA’s configuration enables it to interact with the aromatase active site, hindering substrate aromatization. Indeed, different studies have suggested that UA has the potential to inhibit aromatase activity [120,121].

Gnoatto et al. [122], in their work, reported the inhibitory potency of UA in aromatase enzyme activity. The authors noticed efficient and dose-dependent aromatase inhibition (an IC_50_ value of 32 μM). Through a structure–inhibitory potency relationship study, the pivotal role of C3-OH, C17-COOH, and the size of cycle A of ursolic acid was defined in the inhibition of the enzyme. These data emphasize that the presence of cycle A, along with the free hydroxyl group at C-3 and/or the carboxyl group at C-17 of UA, seems to be necessary for aromatase inhibition, as the esterification of one of these functions led to a significant decrease in inhibitory potency [122]. Thus, the configuration of UA is undoubtedly appropriate for recognizing the active site of the enzyme and hampering substrate aromatization. The structural homology between UA and the aromatase substrate androstenedione further supports this hypothesis.

Moving forward, in 2022, Ma et al. [123] conducted studies demonstrating that UA could interfere with aromatase activity, inhibiting the growth of gastric cancer cells. Through molecular docking simulations, it was found that UA fits into the aromatase binding pocket, forming different hydrophobic interactions and a pi–cation interaction with the amino acids of the binding pocket. The data obtained revealed the specificity of UA in binding aromatase, which resulted in enzyme silencing and gastric cancer (GCa) cell growth suppression [123].

### 2.6. Kaempferol

Kaempferol (KA) is a flavanol present in various plant parts, including seeds, flowers, leaves, and fruits, with a notable abundance in endemic plants of the Mediterranean region. Specifically, kaempferol has been isolated from a range of plant species such as *Rosmarinus officinalis* L., Aloe vera, *Coccinia grandis*, *Equisetum arvense*, Ginkgo biloba, Glycine max, *Laurus nobilis*, *Hypericum perforatum*, *Pinus sylvestris*, *Moringa oleifera*, and *Sambucus nigra* [124].

From a chemical perspective, kaempferol (Table 6) is a tetrahydroxyflavone featuring hydroxy groups located at positions 3, 5, 7, and 4′ [125], and it is a yellow compound. In plants, kaempferol is commonly found in glycosidic form, being conjugated with various sugars such as rutinose, rhamnose, glucose, and galactose [124].

Kaempferol, along with the myriad of other phytochemicals present in rosemary, exhibits a diverse range of biological activities (Table 6). Notably, these include antioxidant, anti-inflammatory, antimicrobial, antitumoral, and antidiabetic properties.

**Table 6:** Two-dimensional structure and biological activity of kaempferol.

Kaempferol		
2D	Biological Activity	References
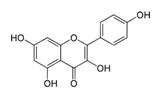	Antioxidant	[126,127]
	Anticancer	[128,129,130,131]
	Anti-inflammatory	[132,133,134]
	Antidiabetic	[135,136]
	Antibacterial	[137,138,139]
	Antidepressant	[140]

Mutoh et al., in their study, tested 14 natural compounds, among them, KA, and noticed that their cancer chemoprotective activity can be linked to the suppression of COX-2 promoter activity in colon cancer cells [141]. Their data suggest that the ability of KA to inhibit COX-2 promoter activity is related to the resorcin moiety [141].

In 2019, Imran et al. [125] reported the antiproliferative activity of KA in human gastric cancer cells (MKN28 and SGC7901) related to promoting autophagy, cell cycle arrest at the G2/M phase, and cell death. The authors linked the induced autophagic cell death to the ability of KA to interfere with different mechanisms, among these, the downregulation of COX-2 [125].

Moving forward, in a study by Song et al. [142], the inhibitory effects of KA on the proliferation of various gastric cancer (GC) cell lines were examined. The study showed that in vitro treatment with KA significantly reduced the expression levels of COX-2, p-AKT, and p-ERK in MKN28 and SGC7901 GC cell lines.

Finally, the ability of KA to inhibit COX-2 activity was also reported by Kaur et al. [143]. In their study, the authors highlighted that KA exhibited inhibitory activity against COX-2, with a remarkable 97.08% inhibition at a concentration of 10 μM and 51.94% at 1 μM.

Lu et al., in their study [144], investigated the effects of daily consumed flavonoids, including KA, on estrogen biosynthesis and their ability to interfere with aromatase, providing significant information. In detail, the authors focused on the effects of kaempferol on cell viability and estrogen biosynthesis in human ovarian granulosa-derived cells (KGNs) correlated with the suppression of aromatase. The results of their investigation revealed that treatment with KA at concentrations of 10 and 50 μM led to significant inhibition of 17-β-estradiol biosynthesis. Specifically, the authors highlighted a 30% aromatase inhibition rate with 10 μM of kaempferol and a 50% aromatase inhibition rate with 50 μM of kaempferol. These data show that KA can directly inhibit the catalytic activity of aromatase in placental microsomes or purified aromatase [144,145]. The authors concluded that the consumption of fruits and vegetables rich in flavonoids such as KA can help to reduce endogenous estrogen levels, aiding in the prevention of estrogen-dependent diseases such as breast cancer [144,145].

## 3. Conclusions

The global rise in cancer incidence highlights an urgent need for the discovery of new anticancer drugs. Despite the availability of several treatments for different types of cancer, no drug has yet been found that is both completely effective and safe. The primary issue stems from the toxicity associated with existing drugs. Nature remains a valuable source of active compounds effective against cancer cells even today. Natural compounds can target different enzymes overexpressed in cancer cells. Given their pivotal role in cancer progression, this review summarizes the most recent advances in the search for natural products acting on aromatase and COX-2.

Throughout history, *Rosmarinus officinalis* L. has been well known as a wide source of phytochemical constituents responsible for several biological activities. Thus, this review aimed to highlight the anticancer properties of some of the phytochemicals contained in *Rosmarinus officinalis* L. We focused on the anticancer properties of carnosic acid, carnosol, rosmarinic acid, betulinic acid, ursolic acid, and kaempferol, which exert their anticancer effects through the modulation of aromatase and COX-2 enzyme activity. The natural compounds mentioned in this review demonstrate promising anticancer activity by acting directly or indirectly on aforementioned enzymes, as evidenced by the reported studies.

In conclusion, this review highlights the potential of the natural constituents of *Rosmarinus officinalis* L. in the search for natural anticancer compounds. Given their remarkable properties, these compounds could serve as a crucial starting point for hit identification and the development of more potent and active derivatives.

## Figures and Tables

**Table 1:** Two-dimensional structure and biological activity of carnosic acid.

Carnosic Acid		
2D	Biological Activity	References
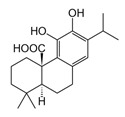	Antioxidant	[53,54]
	Anticancer	[55,56,57,58,59,60]
	Anti-inflammatory	[59,61]
	Anti-lipogenic	[62]
	Antidiabetic	[63]
	Antimicrobial	[54]

**Table 2:** Two-dimensional structure and biological activity of carnosol.

Carnosol		
2D	Biological Activity	References
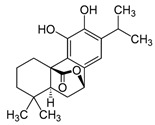	Antioxidant	[65,66]
	Anticancer	[66]
	Anti-inflammatory	[66,67,68]
	Antidiabetic	[69]
	Antimicrobial	[54,70]

**Table 3:** Two-dimensional structure and biological activity of rosmarinic acid.

Rosmarinic Acid		
2D	Biological Activity	References
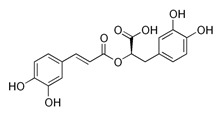	Antioxidant	[71,73,74,75,76]
	Anticancer	[77,78,79,80,81]
	Anti-inflammatory	[82,83,84,85,86]
	Antibacterial	[87]
	Antidiabetic	[88]
	Antiviral	[89,90]
	Neuroprotective	[91,92]
	Hepatoprotective	[72]

**Table 4:** Two-dimensional structure and biological activity of betulinic acid.

Betulinic Acid		
2D	Biological Activity	References
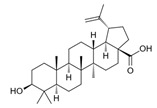	Anticancer	[95,96]
	Anti-inflammatory	[97,98]
	Antiplasmodial	[99]
	Antiviral	[100,101]
	Antidiabetic	[93]

## Data Availability

The data are contained within the article.
